# Accurate prediction of nuclear receptors with conjoint triad feature

**DOI:** 10.1186/s12859-015-0828-1

**Published:** 2015-12-03

**Authors:** Hongchu Wang, Xuehai Hu

**Affiliations:** 1 Department of Mathemaitcs, South China Normal University, Guangzhou, 510631 P.R. of China; 2 College of Informatics, Hubei Key Laboratory of Agricultural Bioinformatics, Huazhong Agricultural University, Wuhan, 430070 P.R. of China

**Keywords:** Nuclear receptors, Conjoint triad feature, Chaos game representation, Amino acid composition, Support vector machine

## Abstract

**Background:**

Nuclear receptors (NRs) form a large family of ligand-inducible transcription factors that regulate gene expressions involved in numerous physiological phenomena, such as embryogenesis, homeostasis, cell growth and death. These nuclear receptors-related pathways are important targets of marketed drugs. Therefore, the design of a reliable computational model for predicting NRs from amino acid sequence has now been a significant biomedical problem.

**Results:**

Conjoint triad feature (CTF) mainly considers neighbor relationships in protein sequences by encoding each protein sequence using the triad (continuous three amino acids) frequency distribution extracted from a 7-letter reduced alphabet. In addition, chaos game representation (CGR) can investigate the patterns hidden in protein sequences and visually reveal previously unknown structure. In this paper, three methods, CTF, CGR, amino acid composition (AAC), are applied to formulate the protein samples. By considering different combinations of three methods, we study seven groups of features, and each group is evaluated by the 10-fold cross-validation test. Meanwhile, a new non-redundant dataset containing 474 NR sequences and 500 non-NR sequences is built based on the latest NucleaRDB database. Comparing the results of numerical experiments, the group of combined features with CTF and AAC gets the best result with the accuracy of 96.30 % for identifying NRs from non-NRs. Moreover, if it is classified as a NR, it will be further put into the second level, which will classify a NR into one of the eight main subfamilies. At the second level, the group of combined features with CTF and AAC also gets the best accuracy of 94.73 %. Subsequently, the proposed predictor is compared with two existing methods, and the comparisons show that the accuracies of two levels significantly increase to 98.79 % (NR-2L: 92.56 %; iNR-PhysChem: 98.18 %; the first level) and 93.71 % (NR-2L: 88.68 %; iNR-PhysChem: 92.45 %; the second level) with the introduction of our CTF-based method. Finally, each component of CTF features is analyzed via the statistical significant test, and a simplified model only with the resulting top-50 significant features achieves accuracy of 95.28 %.

**Conclusions:**

The experimental results demonstrate that our CTF-based method is an effective way for predicting nuclear receptor proteins. Furthermore, the top-50 significant features obtained from the statistical significant test are considered as the “intrinsic features” in predicting NRs based on the analysis of relative importance.

**Electronic supplementary material:**

The online version of this article (doi:10.1186/s12859-015-0828-1) contains supplementary material, which is available to authorized users.

## Background

Nuclear receptors (NRs) are members of a large family of ligand-inducible transcription factors that regulate gene expressions involved in numerous physiological phenomena. These physiological phenomena cover many aspects of multicellular organisms’ lives, including embryogenesis, homeostasis, cell growth and death [1]. Different from cell surface receptors which have strong affinities with water-soluble peptide hormones and growth factors, NRs mostly bind to lipophilic hormone ligands, such as steroids, retinoids, thyroid hormones, vitamin D_3_ and so forth. These fat-soluble ligands can get into cytoplasm through lipid bilayer of cell membranes, and bind to NRs. Furthermore, the resulting allosteric ligand-protein complexes get into cell nucleus and regulate expressions of target genes [1].

All NRs are modular proteins which share common structure organizations. They mostly have 6 (or 5) functional protein domains, including N-terminal A/B domain, DNA-binding domain (DBD, C domain), D domain, ligand-binding domain (LBD, E domain) and F domain of C-terminal end [2]. The N-terminal A/B domain contains at least one activation function 1 region (AF-1) which can operate autonomously and several varied autonomous transactivation domains (AD). It has not now been reported about the crystal structure of A/B domains, which possibly are involved in post-translational modification according to the report [3]. The most conserved domain is DBD, which acts as a central role of binding to specific DNA sequences. Several crystal structures of DBDs are reported, and they usually contain two typical cysteine-rich zinc finger motifs [4, 5]. The P box in the first zinc finger determines the DNA-binding sequence specificity through a short AGGTCA motif. In addition, the D domain contains the nuclear localization signal (NLS) and severs as a hinge between the DBD and the LBD, permitting the DBDs and LBDs to adopt different conformations under hormone activation. Among all the domains, the largest domain is LBD, whose 3D structure is moderately conserved and comprises 12 α-helices and a β-turn [6]. In general, behind helix 3 and in the front of helices 7 and 10, LBD contains at least one ligand-binding pocket, which enables the binding of ligands. Ligand binding will induce a conformational change in LBD of NRs. Furthermore, agonists and antagonists will lead to distinct structural alterations of nuclear receptor LBDs [7]. NRs may or may not contain the F domain, whose structure and function remain unknown [2].

Based on aforementioned six (or five) domains, NRs can perform their function through typical features of domains. They can bind to ligands at the LBD, leading to the allosteric change of their 3D structures. As a result, stronger affinities with chromatin will be made by these conformational changes, which allow NRs to bind to DNA through the DBD. Agonist which acts as activated ligand will enhance the expression of the target gene, whereas antagonist which severs as depressing ligand will silence the gene expression. These specific abilities of regulating gene expressions imply that since NRs are related to major human diseases, such as breast cancer, diabetes, osteoporosis and so on, they are promising pharmacological targets [2]. Basically, NRs are the largest family of hormone receptors, comprising 49 genes in the human genome [8]. According to statistics, about 13 % of marketed drugs target NRs, which are among the one of most frequent targets of therapeutic drugs [9].

Conventional methods for identifying non-annotated proteins are experimental means, such as X-ray crystallography or NMR spectroscopy and so on. These effective techniques provide a detailed 3D structure of a protein for helping understand its function [4–6]. With the absence of experiment conditions, researchers may choose to run a standard basic local alignment search tool (BLAST) [10] to identify a protein to be NR based on the conserved motifs comprising two zinc fingers of the DNA-binding domain [1]. However, NRs are divided into eight classes according to their ligand binding, DNA binding, and dimerization properties [1, 8]. The search tool, such as BLAST, cannot identify subfamilies of NRs [11] because different classes of NRs share low sequence similarities. Therefore, it is essential to develop novel methods to recognize NRs and their subfamilies.

An alternative way to identify NRs is to develop computational methods. With the rapid development of large-scale genome and proteome sequencing project, huge amounts of biological data begin to accumulate. In the area of NRs, the NucleaRDB is a molecular class-specific information system that collects, combines, validates and disseminates large amounts of heterogeneous data on nuclear hormone receptors [8, 12]. The collection of all these data provides possibilities to develop computational methods for predicting the function of NR proteins by their primary sequences. According to the latest release of NucleaRDB (July 01, 2011 - Version 11.7.1), the data are grouped into eight families or classes based on their ligand binding, DNA binding, and dimerization properties of NRs [8]. The eight families are (1) Thyroid hormone like, (2) HNF4-like, (3) Estrogen like, (4) Nerve Growth factor IB-like, (5) Fushi tarazu-F1 like, (6) Germ cell nuclear factor like, (7) Knirps like, and (8) DAX like. These NRs families and their structural features are closely correlated with their function [11], and it would be significant to develop a powerful computational method to classify NRs into particular families for the purpose of understanding their biological function and their potential as future drug targets.

In 2004, an early attempt for predicting NRs and their subfamilies was performed by Bhasin and Raghava based on amino acid composition (AAC) and dipeptide composition (DC) features [11]. Gao et al. [13] developed a feature selection approach to identify relevant features, and a reduced feature subset containing 30 features (18 AACs and 12 DCs) resulted in an improved overall accuracy. In the same year, Gao et al. employed pseudo amino acid composition (PseAA) for predicting and recognizing NRs using support vector machines (SVM) [14]. In 2011, Wang et al. [15] integrated various types of features, such as AAC, DC, complexity factor (CF) and fourier spectrum components (FSC), to represent protein sequences as 881-dimensional vectors. Thus, these sequence-derived features were put into fuzzy K nearest neighbor (FKNN) classifier to identify NRs and their families. Subsequently, Xiao et al. [16] constructed a predicting model based on physical-chemical matrix via a series of auto-covariance and cross-covariance transformations, and resulting predictor achieved higher accuracy rates of recognition on the same dataset [15]. Recently, a proteome-scale two level predicting method, named “NRfamPred”, was developed based on dipeptide composition [17].

Here, we develop an integrated model by employing conjoint triad feature (CTF) and chaos game representation (CGR) to give an appropriate numerical representation of nuclear receptor protein sequence. Originally, CTF was used for prediction of protein-protein interaction (PPI) as important features of protein sequences and achieved excellent performance [18]. Whereafter, CTF was extended to represent protein sequence for identifying RNA-protein interaction (RPI) [19, 20] and became a popular method for suitable representation of protein sequence [21–24]. On the other hand, in 1990, Jeffrey [25] proposed the chaos game representation (CGR) of DNA sequences, and CGR method could excavate hidden patterns in sequences. Subsequently, CGR method of DNA sequences was extended to represent protein sequences by Basu et al. [26], who used CGR algorithm to generate protein sequence by virtue of a 12-sided regular polygon. Each vertex of polygon represented a group of amino acid residues according to conservative substitutions. The authors claimed that CGR had the potential to reveal the evolutionary and functional relationships even between the proteins with no significant sequence homology. Up to present, CGR method has achieved many applications and attracted increasing studies in the area of bioinformatics [27–30].

At present, it is widely believed that the features for input vector of support vector machine (SVM) directly determined the efficiency of prediction model. So far no report yet has been published about CTF, CGR together with AAC as features to predict NRs. In this paper, we will present a CTF-based method, which is proposed to improve the accuracy of the classification of NRs.

## Methods

### Dataset

There are several well known datasets for identify NRs and their subfamilies in the literatures before, such as D282 [11, 13, 14] and D159 [15, 16]. According to the latest information in NucleaRDB website (http://www.receptors.org/nucleardb) and recent publication [8], NucleaRDB updated its contents and information on July 01, 2011. The updated database added some recent-published sequences and structures of NRs, many of which are not been included in D282 and D159 (Table 2). Take more information into consideration, a new dataset was built from the latest version of NucleaRDB in this report. The newly updated NucleaRDB classified all the NRs into eight main families, (1) NR1: thyroid hormone like, (2) NR2: HNF4-like, (3) NR3: estrogen like, (4) NR4: nerve Growth factor IB-like, (5) NR5: fushi tarazu-F1 like, (6) NR6: germ cell nuclear factor like, (7) NR7: knirps like, and (8) NR8: DAX like. All the protein sequences of eight subfamilies were downloaded (detailed information can be found in Table 2).

**Table 1:** Dataset

Dataset	Numbers of NRs	Numbers of Non-NRs
Training Dataset	474	500

To reduce the homology bias of prediction, a redundancy reduction procedure was performed on this dataset by CD-HIT program [31], and a cutoff threshold of 60 % was imposed to exclude those proteins from the benchmark datasets that have equal to or greater than 60 % sequence identity to any other in a same subset. Usually, a cutoff threshold of 25 % was recommended [32–34]. However, such a stringent criterion deduces that number of proteins would be too few to have statistical significance, so the cutoff threshold of 60 % is adopted in this study. As a result, the new dataset contains 474 NR sequences in total. On the other hand, to estimate the ability of the present method in discriminating NRs from non-NRs, a negative dataset containing 500 non-NRs sequences were collected from D159 [15]. Our final training set (denote by D474) contains 474 NR sequences and 500 non-NR sequences (Tables 1, 2), which can be downloaded in the Additional file 1.

**Table 2:** The detailed GPCRs subfamilies of dataset

NRs family	Subset	Number of proteins from NucleaRDB	Number of proteins after CD-HIT (cut off threshold 0.6)	D159 (cut off threshold 0.6)	D282 (cut off threshold 0.9)
Thyroid hormone like	NR1	1172	162	50	114
HNF4-like	NR2	736	140	36	72
Estrogen like	NR3	704	82	37	75
Nerve Growth factor IB-like	NR4	119	23	7	-
Fushi tarazu-F1 like	NR5	151	29	12	21
Germ cell nuclear factor like	NR6	41	7	5	-
Knirps like	NR7	47	21	12	-
DAX like	NR8	46	10	-	-
Overall		3016	474	159	282

### Sample representation

For our computational approach, each protein is represented as a numerical vector, so as to be put into SVM for classification. Actually, a number of methods were used to extract information from protein sequences, for example, amino acid composition (AAC) was used to transform NR sequences into 20-dimension numerical vectors [11]. Meanwhile, in order to extract the information of sequence order, dipeptide composition (DC) was proposed to represent NR sequences by 400-dimension vectors, which captured local-order information and had been reported to improve classifications [11]. In addition, Gao et al. [14] used the concept of Chou’s pseudo amino acid composition to represent each protein sequence by numerical features, which reflected a protein’s overall sequence pattern. Recently, a web server called Pse-in-One [35] was established, which can generate various protein features to construct the predictor. Based on all works mentioned above, here three kinds of feature-derived methods, AAC, CTF, CGR, are employed to capture pivotal information of NR sequences.

### Amino acid composition

Amino acid composition (AAC) was the most popular and also simplest way to represent protein sequences, and it is believed to be the fundamental features to perform protein prediction problems.

More precisely, a protein sequence P with L amino acid residues can be expressed as:

$$ P={R}_1{R}_2{R}_3{R}_4{R}_5\cdots {R}_L. $$

The AAC of a protein is defined as the normalized frequency of each amino acid in that protein; i.e.,

$$ AAC={\left[{f}_1,{f}_2,{f}_3,\cdots, {f}_{20}\right]}^T, $$

where ${f}_i=\frac{n_i}{L}$, and *n*_*i*_ is the occurrence number of the *i*-th amino acid with each *i*(*i* = 1, ⋯, 20).

### Conjoint triad feature

Conjoint triad feature (CTF) was originally used to transform protein sequences into 343-dimension numerical vectors for successfully predicting PPI [18], and was extended to predict RPI [19, 20], enzyme function [21], functional related proteins [23]. CTF clustered 20 amino acids into seven classes ({AGV}, {ILFP}, {YMTS}, {HNQW}, {RK}, {DE}, {C}) according to their dipoles and volumes of the side chains [18]. Subsequently, they regarded any three continuous amino acids as a unit. It is worthy to note that the triads can be categorized according to the classes of amino acids, i.e., triads composed by three amino acids belonging to the same classes can be treated identically. Finally, CTF counts the frequencies of each triad type. By this way, each protein sequence is represented by a 343 (7 × 7 × 7) dimensional vector.

More precisely, a protein sequence P with L amino acid residues can be expressed as:

$$ P={R}_1{R}_2{R}_3{R}_4{R}_5\cdots {R}_L. $$

Then we successively consider sliding windows with continuous three residues *R*_1_ *R*_2_ *R*_3_, *R*_2_ *R*_3_ *R*_4_, *R*_3_ *R*_4_ *R*_5_, ⋯, *R*_*L* − 2_ *R*_*L* − 1_ *R*_*L*_. The CTF of a protein is defined as the normalized frequency of the corresponding 3-mer in that protein; i.e.,

$$ CTF={\left[{f}_1,{f}_2,{f}_3,\cdots, {f}_{343}\right]}^T, $$

where ${f}_i=\frac{n_i}{L-2}$, and *n*_*i*_ is the occurrence number of the *i*-th triad type of all continuous three residues with each *i*(*i* = 1, ⋯, 343). More detailed description for the CTF can be found in the following literatures [18, 23].

### Chaos game representation

**Fig. 1:**
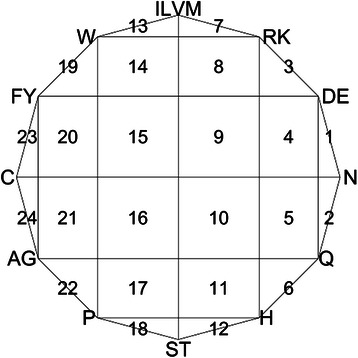
CGR picture. The segments labeled serially with numbers 1–24. (Also can be found in the reference ([30])

The chaos game representation (CGR) algorithm of proteins is first proposed by Basu et al. [26]. The algorithm of CGR picture drawing is listed as below:

Step 1. Draw a 12-sided regular polygon, and each vertex represents a kind group of amino acids (Fig. 1.);

Step 2. Pick the center of polygon *P*_*0*_ to be the initial point;

Step 3. Given a protein sequence with length *N*, we draw *N* points in the polygon by the following way: In turn we read alphabet from the protein sequence, since each read belongs to one group of amino acids, then we determine a certain vertex of polygon and draw the midpoint between initial point *P*_*0*_ and the chosen vertex. After finishing drawing one point, we set it to be the new initial point, and we can draw *N* points with such iteration.

More precisely, if we denote *P*_0_(0, 0) as the center of the polygon and *V*_1_(1, 0) as the first vertex of the polygon, we can easily get coordinates of the other eleven vertexes with the following formula:

1 $$ \left\{\begin{array}{c}\hfill {V}_k(x)= \cos \frac{k-1}{6}\pi \hfill \\ {}\hfill {V}_k(y)= \sin \frac{k-1}{6}\pi \hfill \end{array}\right.\kern1em k=2,\;3,\cdots,\;12. $$

Then we compute coordinates of each CGR point as follows:

2 $$ \left\{\begin{array}{c}\hfill CG{R}_i(x)=\frac{1}{2}\left(CG{R}_{i-1}(x)+{V}_i(x)\right)\hfill \\ {}\hfill CG{R}_i(y)=\frac{1}{2}\left(CG{R}_{i-1}(y)+{V}_i(y)\right)\hfill \end{array}\right.\kern1em i=1,\;2,\cdots,\;N, $$

where *CGR*_*i*_(*x*, *y*) refers to the coordinate of the *i*-th point drawn in the CGR picture, and *V*_*i*_(*x*, *y*) represents the coordinate of chosen vertex by the *i*-th read (each read determines a certain vertex of polygon).

The CGR algorithm can generate an image that contains fractal structure and visually reveal previously unknown structure information for each concatenated amino acid sequences. Furthermore, for the sake of operable mathematical classification, a mathematical characterization of the CGR picture will be needed. We extract the frequency information of each segment by dividing the 12-sided polygon into 24 segments (grids), which are labeled serially with numbers 1–24, as shown in Fig. 1.

For each segment, i.e. *S*_*k*_, *k* = 1, 2, ⋯, 24, we denote by *L*_*k*_, *k* = 1, 2, ⋯, 24 the number of points which fall into *L*_*k*_. The points falling on boundaries of adjacent segments should be counted in any one of the neighboring segment. Then set

3 $$ {D}_k=\frac{L_k}{N},k=1,2,\cdots, 24, $$

where *N* is the length of amino acid sequence. From the above CGR and segment-counting algorithm, we find that each amino acid sequence induces a 24-dimensional vector (*D*_1_, ⋯, *D*_24_).

### Support vector machines

A support vector machine (SVM) performs a nonlinear mapping of the input vector x from the input space, the (a positive integer) dimensional euclidean space, into a higher dimensional Hilbert space, where the mapping is determined by the kernel function. It finds the Optimal Separating Hyper plane (OSH) in the space H corresponding to a non-linear boundary in the input space. For a given data set, only the kernel function and the regularity parameter C must be selected. A complete description to the usage of SVMs for pattern recognition could be found in [36]. In this study, the RBF kernel function (with a parameter *γ*) is adopted and the implementation of SVM is based on LibSVM 3.17, which is an open source that can be downloaded in the website: http://www.csie.ntu.edu.tw/~cjlin/libsvm/index.html.

### Evaluation of the prediction performance

Usually, in statistical prediction, the following three criteria are often used to examine a predictor for its effectiveness in practical application: self-consistency test (re-substitution test), subsampling (K-fold cross-validation) test and jackknife test [37]. Particularly, the jackknife test often can be used to examine a predictor for its effectiveness in practical application [37] because the jackknife test is deemed the most rigorous one that can exclude the memory effects during the entire testing process and can always yield a unique result for a given dataset, as elucidated in [38] and demonstrated by [32]. In this paper, on the one hand, when comparing with other methods, we adopt the jackknife test following the original test method. On the other hand, to test the performance of our hybrid method, we choose 10-fold cross-validation due to the new larger dataset.

Generally, the performance of the prediction method is measured by sensitivity (Sens), specificity (Spec), accuracy (Acc) and Matthew’s correlation coefficient (MCC) value, calculated as:

5 $$ \left\{\begin{array}{l} Sens=\frac{TP}{TP+FN}\\ {} Spec=\frac{TN}{TN+FP}\\ {}Acc=\frac{TP+TN}{TP+FP+TN+FN}\\ {}MCC=\frac{TP\times TN-FP\times FN}{\sqrt{\left(TP+FN\right)\left(TP+FP\right)\left(TN+FP\right)\left(TN+FN\right)}}\end{array}\right. $$

where TP means the number of true positives (NRs predicted as NRs) in one experiment, FN means the number of false negatives (NRs predicted as non-NRs), TN means the number of true negatives (non-NRs predicted as non-NRs), FP means the number of false positives (non-NRs predicted as NRs). Additionally, to test the balance between true positive rate and false positive rate, we also draw the receive operating characteristic (ROC) curves and compute the corresponding the area under the curve (AUC) values (The AUC for a perfect classifier is 1, and for a random classifier is 0.5).

Moreover, for the second level of multi – class classification problem, in order to compute the predicting performance of each class, we follow the evaluation criteria described in [39]. Firstly, four indexes of each subfamily are computed based on Equation 6:

6 $$ \left\{\begin{array}{l}TP(i)={N}^{+}(i)-{N}_{-}^{+}(i)\\ {}TN(i)={N}^{-}(i)-{N}_{+}^{-}(i)\\ {}FP(i)={N}_{-}^{+}(i)\\ {}FN(i)={N}_{+}^{-}(i)\end{array}\right.,i=1,2,\dots, 8. $$

where *N* ^+^(*i*) is the total number of the samples in the subset *NRi*, whereas *N*_−_^+^(*i*) is the number of samples in *NRi* that are incorrectly predicted belonging to the other subsets, and *N* ^−^(*i*) is the total number of samples in all of the other subsets, whereas *N*_+_^−^(*i*) is the number of samples that are incorrectly predicted belonging to *NRi*. Subsequently, the performance of predicting method about each subfamily is evaluated by:

7 $$ \left\{\begin{array}{l} Sens(i)=\frac{TP(i)}{TP(i)+FN(i)}\\ {} Spec(i)=\frac{TN(i)}{TN(i)+FP(i)}\\ {}Acc(i)=\frac{TP(i)+TN(i)}{TP(i)+FP(i)+TN(i)+FN(i)}\\ {}MCC(i)=\frac{TP(i)\times TN(i)-FP(i)\times FN(i)}{\sqrt{\left(TP(i)+FN(i)\left)\left(TP(i)+FP(i)\right)\left(TN(i)+FP(i)\right)\right(TN(i)+FN(i)\right)}}\end{array}\right.,i=1,2,\cdots 8. $$

## Results and discussion

### Predicting NRs and their subfamilies

**Fig. 2:**
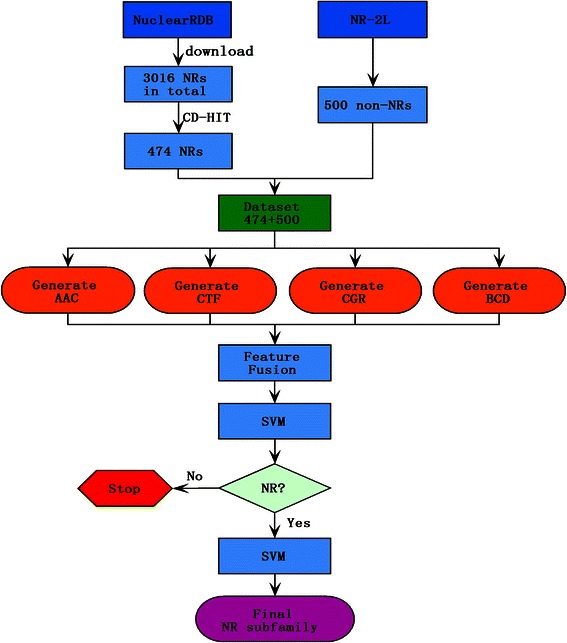
Flowchart to describe the operation process

Firstly, this work focuses on how to seek the best combinations of three groups of feature-derived methods, i.e. AAC, CTF, CGR, to predict nuclear receptors (NRs) and their subfamilies. At the first level, an un-annotated protein is predicted to be either an NR or a non-NR. If it is classified as a NR, it will be further put into the second level, which will classify a NR into one of the eight subfamilies. The detailed flowchart can be found in Fig. 2.

In order to seek the optimal combined features in the feature space, a series of comparative experiments are carried on via 10-fold cross-validation test. More precisely, all the protein sequences are randomly divided into ten groups for the following ten folds, and in each fold, one group is used for testing and other nine groups are used for training. Subsequently, a SVM classifier is trained by using inputting feature vectors and class labels (1 for NR; 0 for non-NR) extracted from the training dataset.

The numerical experiments are designed on seven groups of feature sets. Feature set 1: AAC features (20-dimensional); Feature set 2: CGR features (24-dimensional); Feature set 3: CTF features (343-dimensional); Feature set 4: AAC and CGR features (20 + 24 = 44-dimensional); Feature set 5: AAC and CTF features (20 + 343 = 363-dimensional); Feature set 6: CGR and CTF features (24 + 343 = 367-dimensional); Feature set 7: AAC together with CGR and CTF features (20 + 24 + 343 = 387-dimensional).

**Fig. 3:**
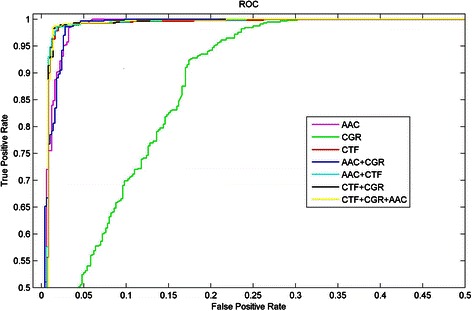
Receiver operating characteristic (ROC) curves for NRs predictions. ROC curves illustrate the trade-off between true positive rate and false positive rate for SVM classifiers, by using seven different groups of feature combinations on new dataset D474

The detailed results which include average values of Sens, Spec, Acc, MCC and AUC in identifying the NR proteins from non-NR proteins are listed in Table 3. From Table 3, for the first level, the average Accs range from 0.8511 to 0.9630, and the average MCCs range from 0.7022 to 0.9261, and the average AUCs range from 0.9290 to 0.9923. Particularly, Feature set 5, i.e. CTF + AAC features, performs the best results, and the average Acc achieves 96.30 % with the optimal parameters *γ* = 0.1899, *C* = 10.1197. Additionally, ROC curves of all seven different feature sets are shown in Fig. 3.

**Table 3:** Results in identifying the NR proteins from non-NR Proteins

Feature set	Dimension	Sens	Spec	Acc	MCC	AUC
AAC	20	0.9388	0.9320	0.9353	0.8706	0.9923
CGR	24	0.8567	0.8460	0.8511	0.7022	0.9290
CTF	343	0.9346	0.9880	0.9620	0.9240	0.9920
AAC + CGR	44	0.9388	0.9240	0.9312	0.8624	0.9923
AAC + CTF	363	0.9451	0.9800	0.9630	0.9261	0.9923
CTF + CGR	367	0.9409	0.9820	0.9620	0.9246	0.9915
CTF + CGR + AAC	387	0.9409	0.9800	0.9610	0.9220	0.9914

(10-fold cross-validation test)

The results in identifying eight main NR families are listed in Table 4, from which we could find that the overall Senss range from 0.6772 to 0.9473, and the overall MCCs range from 0.6311 to 0.9397. Particularly, Feature set 5, CTF + AAC features, performs the best results, and the overall Sens achieves 94.73 % and overall MCC value achieves 0.9397.

**Table 4:** Success rates in identifying eight main NR families

Feature set	Dimension	Overall Sens	Overall MCC	Gamma	C
AAC	20	0.7173	0.6769	2.0231	71.8882
CGR	24	0.6772	0.6311	1.0098	77.9671
CTF	343	0.9430	0.9349	0.0192	11.0849
AAC + CGR	44	0.7806	0.7492	2.5595	13.0576
AAC + CTF	363	0.9473	0.9397	0.0159	10.3440
CTF + CGR	367	0.9409	0.9325	0.0015	104.92
CTF + CGR + AAC	387	0.9409	0.9325	0.0138	11.6455

(10-fold cross-validation test)

Comparing the predicting results of different combinations of features, it is worthy to note three important phenomena. Firstly, Feature set 5, CTF + AAC features, achieves the best performance both in the first level and in the second level, which means that the impact of jointly considering CTF and AAC features is excellent. Secondly, Feature set 3, CTF features, surprisingly achieves the second best performance after Feature set 5, which implies that CTF features alone may achieve relatively good results. Particularly, if we compare the predicting performances between Feature set 3 and Feature set 6 (Table 3 and Table 4), we find that the overall acc unexpectedly reduces from 0.9430 to 0.9409 (Table 4) or remains equal (Table 3) when CGR features are added to CTF features, demonstrating that CGR features cannot provide useful helps in predicting NRs and their subfamilies. Thirdly, the differences between Feature set 5 and Feature set 3 are rather small, indicating that AAC features contribute little to predictions.

Those above results lead us to conclude that CTF is an important feature in prediction of NRs and their subfamilies. When feature combinations are AAC (or CGR) with CTF feature lost, the average Acc of first level and second level are at most 93 % and 71 % respectively, whereas the average accuracy of first level and second level have promoted up to 96 % and 94 % respectively when CTF feature is added.

At the second level, for the purpose of investigating the detailed predicting performances of each subfamilies between the two best feature set (Feature set 5 and Feature set 3), we list more detailed predicting information which includes specific values of Sens, Spec, Acc, MCC in each subfamilies in Table 5. It is noteworthy that Feature set 3 and Feature set 5 both perform satisfactory results, and the overall Sens achieve 0.9430 and 0.9473 respectively, which also illustrates that among all the 474 NRs, 447 NRs and 449 NRs are correctly classified into their original subfamilies respectively.

**Table 5:** Predicting performance in identifying eight main NR families based on Feature set 3 and Feature set 5

NR Subfamily	CTF				CTF + AAC			
	Sens(i)	Spec(i)	Acc(i)	MCC(i)	Sens(i)	Spec(i)	Acc(i)	MCC(i)
NR1	158/162 = 0.9753	0.9519	0.9599	0.9135	158/162 = 0.9753	0.9551	0.9620	0.8966
NR2	132/140 = 0.9429	0.9700	0.9620	0.9091	133/140 = 0.95	0.9731	0.9663	0.9189
NR3	77/82 = 0.9390	0.9949	0.9852	0.9479	78/82 = 0.9512	0.9949	0.9873	0.955
NR4	20/23 = 0.8696	1	0.9937	0.9294	20/23 = 0.8696	1	0.9937	0.9294
NR5	27/29 = 0.9310	1	0.9958	0.9627	27/29 = 0.9310	1	0.9958	0.9627
NR6	5/7 = 0.7143	1	0.9958	0.8434	5/7 = 0.7143	1	0.9958	0.8433
NR7	20/21 = 0.9524	1	0.9979	0.9748	20/21 = 0.9524	1	0.9979	0.9748
NR8	8/10 = 0.8	1	0.9958	0.8925	8/10 = 0.8	1	0.9958	0.8340
Overall	447/474 = 0.9430	0.9919	0.9858	0.9349	449/474 = 0.9473	0.9925	0.9868	0.9397

(10-fold cross-validation test)

### Comparisons with other methods at the first level

Many existing methods have classified NRs at a single level. In order to explain the superiority of our hybrid methods, we implement our algorithms on the same dataset (D159, 159 NRs, seven subfamilies) in NR-2L [15] and iNR-PhysChem [16] via the same test method-jackknife test. As a result, we list the detailed comparisons between our methods (Feature set 1-7) and existing methods (NR-2L, iNR-PhysChem) in Table 6.

**Table 6:** Comparisons with NR-2L and iNR-PhysChem at a single level (jackknife test)

Feature	Dimension	Acc	MCC	Independent test dataset
AAC	20	0.9348	0.8288	0.9504
CGR	24	0.8847	0.7693	0.8268
CTF	343	0.9863	0.9625	0.9831
AAC + CGR	44	0.9439	0.8457	0.9410
AAC + CTF	363	0.9879	0.9667	0.9878
CTF + CGR	367	0.9863	0.9727	0.9850
CTF + CGR + AAC	387	0.9848	0.9583	0.9878
NR-2L	881	0.9256	0.8500	0.9803
iNR-PhysChem	1000	0.9818	0.9600	-

From Table 6, as was expected, Feature set 5 again achieves the best predicting performances, which includes Acc value with 98.79 % and MCC value with 0.9667, higher than 92.56 %, 0.8500 from NR-2L [15] and 98.18 %, 0.9600 from iNR-PhysChem respectively. As same as before, Feature set 3 also achieves the second best results and the differences between Feature set 3 and Feature set 5 are also very small. Another noteworthy thing is that the predicting performances of Feature set 3,5,6,7 from our methods are all better than NR-2L and iNR-PhysChem. The comparisons above indicate that our method has achieved a higher overall accuracy on the same benchmark datasets than some previous methods.

### Comparisons with other methods at the second level

We also make comparison with NR-2L [15] and iNR-PhysChem [16] developed on dataset D159 (159 NRs, seven subfamilies) at the second level. NR-2L is the first classifier for predicting NRs at two levels with seven subfamilies. We implement our method on D159 at the second level via the same test method-jackknife test. All the detailed results and comparisons between our method (Feature set 3) and existing methods (NR-2L, iNR-PhysChem) are listed in Table 7.

**Table 7:** Comparisons with NR-2L and iNR-PhysChem at the second level (jackknife test)

NR Subfamily	CTF		NR-2L		iNR-PhysChem	
	Sens(i)	MCC(i)	Sens(i)	MCC(i)	Sens(i)	MCC(i)
NR1	49/50 = 0.9800	0.9029	43/50 = 0.8600	0.88	47/50 = 0.9400	0.87
NR2	32/36 = 0.8889	0.8907	31/36 = 0.8611	0.85	35/36 = 0.9722	0.93
NR3	37/37 = 1	0.9660	37/37 = 1.00	0.86	37/37 = 1.00	0.95
NR4	6/7 = 0.8571	0.9228	6/7 = 0.8571	0.70	5/7 = 0.7143	0.84
NR5	10/12 = 0.8333	0.9067	10/12 = 0.8333	0.86	10/12 = 0.8333	0.91
NR6	5/5 = 1	1	5/5 = 1.00	1.00	5/5 = 1.00	1.00
NR0	10/12 = 0.8333	0.9067	9/12 = 0.7500	0.86	8/12 = 0.6667	0.81
Overall	149/159 = 0.9371	0.9266	141/159 = 0.8868	0.87	147/159 = 0.9245	0.91

Predicting results from Table 7 demonstrate that CTF method results in an overall Sens of 93.71 % at the second level of D159 dataset, higher than 88.68 % from NR-2L and 92.45 % from iNR-PhysChem. Significantly, comparing NR-2L and iNR-PhysChem, predicting performance increases five and two percent by using CTF method respectively. These results indicate that the proposed method of this paper outperforms NR-2L and iNR-PhysChem at the second levels.

### NR proteins and non-NR proteins display distinct CTF-feature properties

Above results demonstrate that CTF method shows superiority both in the first level and in the second level when comparing existing methods and other methods. Next, for the propose of investigating “intrinsic features” among CTF features, we perform the statistical test between 474 NR proteins and 500 non-NR proteins for each feature which is taken from 343 CTF features (two-side Wilcoxon rank-sum test). As a result, 279 of the overall 343 features show significant differences between NR proteins and non-NR proteins (*p* < 0.01, each detailed *p*-value can be found in the Additional file 2). Among all the features, the most two significant features are the 35th feature ({C}-{RK}-{AGV}) and 239th feature ({AGV}-{C}-{RK}) (corresponding *p*-values are 2.83 × 10^− 113^, 1.28 × 10^− 109^ respectively). For the convenience of following analysis, we list the names and their corresponding *p*-values of the top 50 significant features in the Table 8. It is noteworthy that the *p*-values of the top-50 significant features are all below 1.00 × 10^− 23^, which means that these top-50 features all display distinct properties between 474 NR proteins and 500 non-NR proteins. It leads us to consider these top- 10 (or top-50) significant features are the “intrinsic features” in identifying NR proteins.

**Table 8:** The top-50 significant features in CTF and their *p*-values

ID	Feature	*p*-value	ID	Feature	*p*-value
1	{C}-{RK}-{AGV}	2.83E-113	26	{HNQW}-{YMIS}-{C}	1.83E-30
2	{AGV}-{C}-{RK}	1.28E-109	27	{ILFP}-{ILFP}-{YMIS}	4.15E-30
3	{C}-{AGV}-{DE}	1.10E-89	28	{AGV}-{ILFP}-{ILFP}	8.51E-30
4	{DE}-{AGV}-{C}	2.48E-89	29	{YMIS}-{ILFP}-{ILFP}	1.25E-29
5	{C}-{RK}-{ILFP}	5.91E-89	30	{YMIS}-{YMIS}-{C}	1.34E-29
6	{C}-{DE}-{AGV}	1.08E-85	31	{YMIS}-{AGV}-{YMIS}	1.70E-29
7	{YMIS}-{C}-{DE}	1.33E-72	32	{RK}-{AGV}-{C}	9.04E-29
8	{RK}-{C}-{ILFP}	3.69E-72	33	{YMIS}-{AGV}-{AGV}	1.34E-28
9	{RK}-{RK}-{C}	2.15E-54	34	{YMIS}-{AGV}-{C}	3.07E-28
10	{AGV}-{C}-{AGV}	1.37E-48	35	{ILFP}-{HNQW}-{DE}	1.03E-27
11	{YMIS}-{C}-{RK}	1.93E-46	36	{HNQW}-{YMIS}-{AGV}	1.21E-27
12	{RK}-{C}-{HNQW}	1.15E-42	37	{RK}-{RK}-{YMIS}	1.93E-27
13	{ILFP}-{RK}-{RK}	3.18E-41	38	{RK}-{YMIS}-{ILFP}	1.06E-26
14	{HNQW}-{RK}-{C}	2.37E-40	39	{RK}-{ILFP}-{ILFP}	2.32E-26
15	{YMIS}-{YMIS}-{YMIS}	7.58E-40	40	{ILFP}-{YMIS}-{YMIS}	3.03E-26
16	{RK}-{AGV}-{ILFP}	5.18E-37	41	{ILFP}-{AGV}-{C}	5.91E-26
17	{HNQW}-{HNQW}-{C}	1.32E-36	42	{RK}-{HNQW}-{C}	1.57E-25
18	{AGV}-{DE}-{RK}	4.68E-36	43	{ILFP}-{YMIS}-{ILFP}	2.26E-25
19	{ILFP}-{ILFP}-{RK}	4.00E-34	44	{DE}-{RK}-{AGV}	2.62E-25
20	{AGV}-{AGV}-{YMIS}	1.25E-33	45	{ILFP}-{YMIS}-{DE}	5.45E-25
21	{C}-{ILFP}-{AGV}	2.43E-33	46	{C}-{AGV}-{AGV}	7.34E-25
22	{YMIS}-{YMIS}-{ILFP}	2.21E-31	47	{YMIS}-{ILFP}-{HNQW}	9.15E-25
23	{ILFP}-{ILFP}-{ILFP}	2.27E-31	48	{C}-{HNQW}-{AGV}	1.05E-24
24	{AGV}-{YMIS}-{YMIS}	4.38E-31	49	{YMIS}-{ILFP}-{YMIS}	3.83E-24
25	{AGV}-{YMIS}-{AGV}	8.37E-31	50	{C}-{HNQW}-{YMIS}	4.09E-24

### Relative importance of significant CTF features

To further verify these top-10 (or top-50) significant features are the “intrinsic features” in identifying NR proteins, we perform a detailed analysis of relative important of these features. Precisely, considering that these top-10 (or top-50) significant features are particularly importance for NR proteins predictions, we ask whether our prediction model could be simplified by using these top-10 (or top-50) features alone.

To answer this question, we adopt a two-direction strategy to demonstrate the importance of these significant features. One is to perform the predictions by using only top-10 (or top-50) features, whereas another is to perform the predictions by using the remaining CTF features with top-10 (or top-50) features (denote by “CTF-10”, or “CTF-50”) taken away. Remarkably, the performance of the simplified (top-50 significant features, Acc = 0.9528) and the full (343 CTF features, Acc = 0.9620) models is not significantly different (Table 9), whereas the difference between the performance of the CTF-50 model (CTF features with top-50 features taken away, 293 features, Acc = 0.9035) and the performance of the full model (343 CTF features, Acc = 0.9620) is obviously large (Table 9). Our findings indicate that the top-50 significant features are truly “intrinsic features” in identifying NR proteins, and we surmise these features contain substantial conserved motif information of NR proteins.

**Table 9:** Relative importance of the top-50 significant features

Feature	Dimension	D159		D474	
		Acc	MCC	Acc	MCC
CTF	343	0.9863	0.9625	0.9620	0.9240
Top-10	10	0.9621	0.9241	0.9312	0.8624
Top-50	50	0.9772	0.9545	0.9538	0.9076
CTF-10	333	0.9681	0.9363	0.9384	0.8768
CTF-50	293	0.9408	0.8816	0.9035	0.8070
NR-2L	881	0.9256	0.8500	-	-
iNR-PhysChem	1000	0.9818	0.9600	-	-

### Further discussion

With the purpose of supporting our method, a further discussion is proposed. The results mentioned in Table 6 and 7 show that our novel method is superior to NR-2L and iNR-PhysChem. Investigates its reason, the CTF method plays a crucial role in predicting NRs. According to reports, amino acid composition (AAC) are simplest but effective features in predicting NRs [11, 13, 14], however, only AAC features are insufficient with a lake of sequence order information. To compensate for this deficiency, CTF- and CGR-based method is proposed in this research. From the results of Tables 3 and 4, the best accuracy achieves in the group with combined features of CTF and AAC. Moreover, the detailed comparisons between different features show an interesting phenomenon. On the one hand, we find that CTF are fundamental features and each group with absence of CTF achieves unsatisfied accuracy from the detailed results of Tables 3 and 4. On the other hand, although only CTF features cannot achieve the best accuracy, the predicting performances of only CTF features are good enough, so that they are already better than the two existing methods (NR-2L and iNR-PhysChem).

Taking above results into consideration, it is worthy to explore the reasons why CTF features are important for predicting NRs. Let us recall what CTF was and the relationship between CTF and prediction of protein-protein interactions (PPIs). In 2007, CTF originally was proposed to solve PPIs prediction problems [18]. The authors took the attitude that PPIs were mostly dominated by electrostatic and hydrophobic interactions between amino acids from interacting proteins, which might be reflected by the dipoles and volumes of the side chains of amino acids, respectively. Subsequently, 20 kinds of amino acids were classified into seven classes based on their dipoles and their volumes of the side chains. The amino acids belong to the same class were considered to have similar electrostatic and hydrophobic properties. Finally, any continuous amino acids were considered as a unit, from which 343 numerical features were extracted based on their conjoint electrostatic and hydrophobic properties. The CTF method based on conjoint electrostatic and hydrophobic properties naturally was extended to study RNA-protein interactions [19, 20] for the reasons that RNA-protein interaction also might be influenced by electrostatic and hydrophobic interactions between amino acid (from protein) and nucleic acid (from RNA) similarly.

In situation of predicting NRs, proteins which probably are considered as NRs mostly are involved in several interactions, including between small molecules (in cytoplasm, through LBD), between other proteins and between DNA (in nucleus, through DBD). All these interactions are related to electrostatic and hydrophobic interactions, which might be the reasons why CTF method can get better performances than other existing methods in this study.

## Conclusions

Nuclear receptors play a vitally important role in many processes of transcriptional regulations. The conjoint triad feature clusters 20 amino acids into seven classes according to their dipoles and volumes of the side chains. Any three continuous amino acids are regarded as a unit, from which 343 features can be extracted. The chaos game representation algorithm presents each protein sequence to a CGR picture with an iterated fractal approach. CGR pictures are divided into different segments, from which 24 quantitative features are extracted by computing the frequencies of points in each of the segments. We combine two factors (CTF, CGR) with amino acid composition as the candidate features which are used to predict NRs and their subfamilies by SVM based on a newly building dataset.

Taking the results into consideration, on the one hand, we can find the highest predicting Acc and MCC value achieve in the combination of CTF and AAC, with the best average accuracy of 96.30 % and MCC value of 0.9261 at the first level by 10-fold cross-validation. At the second level, the combined features of CTF and AAC also get the best overall Sens of 94.73 %. It is noteworthy that only CTF features also achieve the satisfactory results, average accuracy is 96.20 % for the first level and the overall Sens is 94.30 % for the second level. The differences between CTF + AAC features and only CTF features are not significant. These considerable results suggest that CTF method is an effective way to predict NRs and their subfamilies.

Considering the importance of CTF method, we further analyze each feature from CTF method by statistical significant test. As a result, we select the top-50 significant features by ranking the *p*-value of statistical test. At last, a simplified model with only these 50 features is used to predict NRs and achieve average accuracy of 95.28 % (comparing CTF + AAC, 96.30 %). Another remaining feature set with those top-50 significant features taken away (343–50 = 293 dimensional) is designed to predict NRs and the corresponding average accuracy falls to 90.35 %. These analyses of relative importance lead us to conclude that the top-50 significant features are “intrinsic features” for predicting NRs from non-NRs.

Actually, so far several papers addressed the problem of predicting NRs [11, 13–16]. Among them, Wang et al. [15] constructed a predicting model, called NR-2L. They studied several groups of features from the primary sequence and predicted NRs and their subfamilies by Fuzzy K nearest neighbor (FK-NN) classifier both in a non-redundant training set and an independent dataset. Finally, they got the results with accuracy of 92.56 % and of 88.68 % respectively. To compare with the existing methods NR-2L [15] and iNR-PhysChem [16], we predict the same datasets mentioned in NR-2L with our model. We find that accuracies and MCC values significantly increase to 98.79 % (NR-2L: 92.56 %; iNR-PhysChem: 98.18 %) and 93.71 % (NR-2L: 88.68 %; iNR-PhysChem: 92.45 %) with the introduction of our CTF-based method. The comparisons to the previous works demonstrate that our CTF-based method outperforms the existing methods.

On the base of all the above efforts, we conclude that our CTF-based method adds some new contributions in the area of predicting NRs and their subfamilies:

1. New contribution to dataset: Although D159 is an excellent benchmark dataset, it was constructed in 2011 on the base of NucleaRDB release 5.0. Actually, NucleaRDB updated its contents in 2012 with more NR protein sequences added. The dataset which is built in this paper increase NR protein sequences from 159 (D159) to 474, most of which are newly added to the subsequent study.
2. New contribution to methodology: CTF method was originally invented for predicting protein-protein interactions, and it was extended to identify RNA-protein interactions. Although CTF method is not a newly invented method, to our best knowledge, no reports employed it to predict NRs and their subfamilies. In this paper, we employ CTF method to perform such a prediction and obtain some improvements comparing existing methods.
3. New contribution to the feature selection: Furthermore, each component of CTF features is analyzed via the statistical significant test, and the resulting top-50 features (ranking by *p*-value) are considered as the “intrinsic features” in predicting NRs based on the analysis of relative importance.

In conclusion, a CTF-based method is proposed and the detailed results imply that this method is an effective way to predict NRs and their subfamilies. For the future effort, user-friendly and publicly accessible web-servers represent a future direction for developing practically more useful models, simulated methods, or predictors [40-42], and we shall make efforts in our future work to provide a web-server for the method presented in this paper.

## Additional files

Additional file 1:
**Dataset.** (RAR 140 kb)
Additional file 2:
**P-values of CTF features in statistical test bewteen positive samples and negative samples.** (XLS 43 kb)

## References

[1] Mangelsdorf DJ, Thummel C, Beato M, Herrlich P, Schutz G, Umesono K, Blumberg B, Kastner P, Mark M, Chambon P, Evans RM (1995). The nuclear receptor superfamily: the second decade. Cell.

[2] Robinson-Rechavi M, Garcia HE, Laudet V (2003). The nuclear receptor superfamily. J Cell Sci.

[3] Germain P, Staels B, Dacquet C, Spedding M, Laudet V (2006). Overview of Nomenclature of Nuclear Receptors. Pharmacol Rev.

[4] Luisi BF, Xu WX, Otwinowski Z, Freedman LP, Yamamoto KR, Sigler PB (1991). Crystallographic analysis of the interaction of the glucocorticoid receptor with DNA. Nature.

[5] Schwabe JW, Chapman L, Finch JT, Rhodes D (1993). The crystal structure of the estrogen receptor DNA-binding domain bound to DNA: how receptors discriminate between their response elements. Cell.

[6] Bourguet W, Ruff M, Chambon P, Gronemeyer H, Moras D (1995). Crystal structure of the ligand-binding domain of the human nuclear receptor RXR-alpha. Nature.

[7] Bourguet W, Germain P, Gronemeyer H (2000). Nuclear receptor ligand-binding domains: three-dimensional structures, molecular interactions and pharmacological implications. Trends Pharmacol Sci.

[8] Vroling B, Thorne D, McDermott P, Joosten HJ, Attwood TK, Pettifer S, Vriend G (2012). NucleaRDB: information system for nuclear receptors. Nucleic Acids Res.

[9] Overington JP, Al-Lazikani B, Hopkins AL (2006). How many drug targets are there?. Nat Rev Drug Discov.

[10] Altschul SF, Madden TL, Schaffer AA, Zhang J, Zhang Z, Miller W, Lipman DJ (1997). Gapped BLAST and PSI-BLAST: a new generation of protein database search programs. Nucleic Acids Res.

[11] Bhasin M, Raghava GPS (2004). Classification of nuclear receptors based on amino acid composition and dipeptide composition. J Biol Chem.

[12] Horn F, Vriend G, Cohen FE (2001). Collecting and harvesting biological data: the GPCRDB and NucleaRDB information systems. Nucleic Acids Res.

[13] Gao QB, Jin ZC, Ye XF, Wu C, Lu J, He J (2009). Improving the classification of nuclear receptors with feature selection. Protein Pept Lett.

[14] Gao QB, Jin ZC, Ye XF, Wu C, Lu J, He J (2009). Prediction of nuclear receptors with optimal pseudo amino acid composition. Anal Biochem.

[15] Wang P, Xiao X, Chou KC (2011). NR-2L: a two-level predictor for identifying nuclear receptor subfamilies based on sequence-derived features. PLoS One.

[16] Xiao X, Wang P, Chou KC (2012). iNR-PhysChem: a sequence-based predictor for identifying nuclear receptors and their subfamilies via physical-chemical property matrix. PLoS One.

[17] Kumar R, Kumari B, Srivastava A, Kumar M (2014). NRfamPred: A proteome-scale two levelmethod for prediction of nuclear receptor proteins and their sub-families. Sci Rep.

[18] Shen JW, Zhang J, Luo XM, Zhu WL, Yu KQ, Chen KX, Li YX, Jiang HL (2007). Predicting protein–protein interactions based only on sequences information. P Natl Acad Sci USA.

[19] Shao X, Tian Y, Wu L, Wang Y, Jing L, Deng N (2009). Predicting DNA-and RNA-binding proteins from sequences with kernel methods. J Theor Biol.

[20] Muppirala UK, Honavar VG, Dobbs D (2011). Predicting RNA-protein interactions using only sequence information. BMC Bioinformatics.

[21] Wang YC, Wang Y, Yang ZX, Deng NY (2011). Support vector machine prediction of enzyme function with conjoint triad feature and hierarchical context. BMC Syst Biol.

[22] Kshirsagar M, Carbonell L, Klein-Seetharaman J (2012). Techniques to cope with missing data in host-pathogen protein interaction prediction. Bioinformatics.

[23] Lin TW, Wu JW, Chang DT (2013). Combining phylogenetic profiling-based and machine learning-based techniques to predict functional related proteins. PLoS One.

[24] Huang Y, Liu S, Guo D, Li L, Xiao Y (2013). A novel protocol for three-dimensional structure prediction of RNA-protein complexes. Sci Rep.

[25] Jeffrey HJ (1990). Chaos game representation of gene structure. Nucleic Acids Res.

[26] Basu S, Pan A, Dutta C, Das J (1997). Chaos game representation of proteins. Molecular and Modelling.

[27] Yu ZG, Anha V, Lau KS (2004). Chaos game representation of protein sequences based on the detailed HP model and their multifractal and correlation analyses. J Theor Biol.

[28] Yang JY, Peng ZL, Yu ZG, Zhang RJ, Anh V, Wang D (2009). Prediction of protein structural classes by recurrence quantification analysis based on chaos game representation. J Theor Biol.

[29] Liu XL, Lu JL, Hu XH (2011). Predicting thermophilic proteins with pseudo amino acid composition: approached from chaos game representation and principal component analysis. Protein Pept Lett.

[30] Lu JL, Hu XH, Hu DG (2012). A new hybrid fractal algorithm for predicting thermophilic nucleotide sequences. J Theor Biol.

[31] Huang Y, Niu BF, Gao Y, Fu LM, Li WZ (2010). CD-HIT Suite: a web server for clustering and comparing biological sequences. Bioinformatics.

[32] Chou KC, Shen HB (2007). Recent progress in protein subcellular location prediction. Anal Biochem.

[33] Zhu PP, Li WC, Zhong ZJ, Deng EZ, Ding H, Chen W, Lin H (2015). Predicting the subcellular localization of mycobacterial proteins by incorporating the optimal tripeptides into the general form of pseudo amino acid composition. Mol Biosyst.

[34] Lin H, Chen W, Ding H (2013). AcalPred: a sequence-based tool for discriminating between acidic and alkaline enzymes. PLoS One.

[35] Liu B, Liu F, Wang X, Chen J, Fang L, Chou KC (2015). Pse-in-One: a web server for generating various modes of pseudo components of DNA, RNA, and protein sequences. Nucleic Acids Res.

[36] Vapnik V (1998). Statistical Learning Theory.

[37] Chou KC, Zhang CT (1995). Prediction of protein structural classes. Crit Rev Biochem Mol Biol.

[38] Chou KC, Shen HB (2008). Cell-PLoc: a package of Web servers for predicting subcellular localization of proteins in various organisms. Nat Protoc.

[39] Feng PM, Chen W, Lin H, Chou KC (2013). iHSP-PseRAAAC: Identifying the heat shock protein families using pseudo reduced amino acid alphabet composition. Anal Biochem.

[40] Chou KC (2011). Some remarks on protein attribute prediction and pseudo amino acid composition (50th Anniversary Year Review). J Theor Biol.

[41] Liu B, Fang L, Liu F, Wang X, Chen J, Chou KC (2015). Identification of real microRNA precursors with a pseudo structure status composition approach. PLoS One.

[42] Liu B, Liu F, Fang L, Wang X, Chou KC (2015). repDNA: a Python package to generate various modes of feature vectors for DNA sequences by incorporating user-defined physicochemical properties and sequence-order effects. Bioinformatics.

